# Beclin 1 Expression is Closely Linked to Colorectal Carcinogenesis and Distant Metastasis of Colorectal Carcinoma

**DOI:** 10.3390/ijms150814372

**Published:** 2014-08-18

**Authors:** Mei-Ying Zhang, Wen-Feng Gou, Shuang Zhao, Xiao-Yun Mao, Zhi-Hong Zheng, Yasuo Takano, Hua-Chuan Zheng

**Affiliations:** 1 Laboratory Animal Center, China Medical University, Shenyang 110001, China; E-Mails: zhmeiying@hotmail.com (M.-Y.Z.); zhihongzheng@163.com (Z.-H.Z.); 2 Department of Biochemistry and Molecular Biology, College of Basic Medicine, China Medical University, Shenyang 110001, China; E-Mails: xiaogouaeiou@163.com (W.-F.G.); zhaoshuang1235 @163.com (S.Z.); 3 Department of Surgical Oncology, the First Affiliated Hospital of China Medical University, Shenyang 110001, China; E-Mail: maoxiaoyun@126.com; 4 Clinical Research Institute, Kanagawa Cancer Center, Yokohama 241-0815, Japan; E-Mail: ytakano@gancen.asahi.yokohama.jp

**Keywords:** colorectal carcinoma, Beclin 1, tumorigenesis, pathogenesis, prognosis

## Abstract

Beclin 1 participates in development, autophagy, differentiation, anti- apoptosis, neurodegeneration, tumorigenesis and cancer progression. The roles of *Beclin 1* in colorectal carcinogenesis and its subsequent progression are still unclear. Here, the mRNA and protein expression of Beclin 1 were determined in colorectal carcinoma and matched mucosa by Reverse transcriptase-polymerase chain reaction and Western blot. Immunohistochemistry and* in situ* hybridization (ISH) were performed on tissue microarryer with colorectal carcinoma, adenoma and mucosa. The expression of *Beclin 1* mRNA and protein was found to be higher in colorectal carcinoma than matched mucosa by real-time PCR and Western blot (*p* < 0.05). According to the ISH data, *Beclin 1* expression was lower in colorectal non-neoplastic mucosa (NNM) than adenoma and carcinoma (*p* < 0.05). Immunohistochemically, primary carcinoma showed stronger Beclin 1 expression than NNM and metastatic carcinoma in the liver (*p* < 0.05). Beclin 1 protein expression was negatively related to liver and distant metastasis (*p* < 0.05), but not correlated with age, sex, depth of invasion, lymphatic or venous invasion, lymph node metastasis, tumor-node-metastasis (TNM) staging, differentiation or serum carcinoembryonic antigen (CEA) concentration (*p* > 0.05). Survival analysis indicated that Beclin 1 expression was not linked to favorable prognosis of the patients with colorectal carcinoma (*p* > 0.05). Cox’s model indicated that depth of invasion and distant metastasis were independent prognostic factors for colorectal carcinomas (*p* < 0.05). It was suggested that Beclin 1 expression is closely linked to colorectal carcinogenesis and distant metastasis of colorectal carcinoma.

## 1. Introduction

Beclin 1 functions as a scaffold for the formation of autophagosomes and may be a haploin-sufficient tumor suppressor [1]. Beclin 1 has at least three domains within the evolutionarily conserved *C*-terminal such as the coiled-coil and leucine zipper domains [2]. Functionally, Beclin 1 is involved in autophagy activation, inhibition of cell proliferation, tumorigenesis, development and neurodegeneration via the interaction with such cofactors as Atg14L, UVRAG, Bif-1, Rubicon, Ambra1, HMGB, nPIST, VMP1, SLAM, IP(3)R, PINK and survivin to regulate the lipid kinase Vps-34 protein and promote formation of Beclin 1-Vps34-Vps15 core complexes [1,2,3,4]. Beclin 1 can interact with the BH3 receptor domain (hydrophobic grove) of the anti-apoptotic proteins Bcl-2, Bcl-xL, Bcl-w via BH3 amphipathic α-helix, to regulate basal autophagy levels and cell survival [3,4]. This binding can be disrupted by phosphorylation or ubiquitination of Beclin 1 which is controlled by PI3K-III or different ubiquitin ligases [5]. In cervical cancer HeLa cells, Beclin 1 knockdown promoted the cell proliferation, but Beclin 1 overexpression enhanced the autophagy and cell death through the regulation of caspase-9 expression [6]. Reportedly, mitochondria-mediated apoptosis inhibits autophagy at the execution stage subsequent to cytochrome c release through Beclin 1 cleavage induced by caspase-3 and caspase-8 [7,8].

Bi-allelic loss of *Beclin 1* in mice is embryonically lethal and mice with mono-allelic loss of *Beclin 1* have an increased incidence of spontaneous tumorigenesis, including lymphomas, liver and lung cancers [9,10,11]. In human, mono-allelic deletions of *Beclin 1* are frequently observed in sporadic breast cancer [12]. Beclin 1 protein expression is down-regulated in cervical [13], hepatocellular [14] and ovarian carcinomas [15]. Decreased *Beclin 1* mRNA expression has been observed in glioblastoma, high-grade brain tumors [16] and lung cancer [17]. Myung* et al.* [18] reported that Beclin 1 overexpression was independently associated with overall worse survival of the patients who received 5-fluorouracil-based adjuvant therapy. To determine the roles of *Beclin 1* in colorectal carcinogenesis and its subsequent progression, we collected a large number of colorectal mucosa, adenoma, primary and metastatic carcinoma to examine the expression of *Beclin 1* mRNA and protein by different methods, and compared with clinicopathological parameters and survival data of carcinomas.

## 2. Results and Discussion

### 2.1. Similar Beclin 1 Expression on Colorectal Carcinoma Cell Lines

**Figure 1:**
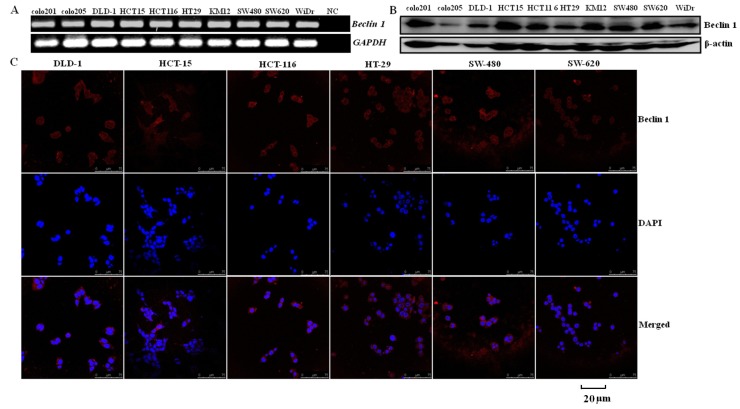
Beclin 1 expression in colorectal carcinoma cells.* Beclin 1* mRNA (160 bp) was detected and showed consistent density in all colorectal carcinoma cell lines with an internal control of *GAPDH* (135 bp) by real time RT-PCR (**A**); Cell lysates were loaded and probed with anti-Beclin 1 antibody with β-actin (42 kDa) as an internal control (**B**); Beclin 1 expression was observed in the cytoplasm of colorectal carcinoma cells by immunofluorescence (Red staining: Beclin 1; blue staining: 4,6-diamino-2-phenyl indole (DAPI)) (**C**). NC, negative control.

*Beclin 1* was similarly detected in all colorectal carcinoma cells at both the mRNA and protein levels (Figure 1A,B). The immunofluorescence staining showed that Beclin 1 protein was distributed in the cytoplasm of DLD-1, HCT-15, HCT-116, HT-29, SW-480 and SW-620 cells (Figure 1C). These findings suggested a similar level of cytosolic Beclin 1 protein in colorectal carcinomas.

### 2.2. Up-Regulated Beclin 1 Expression in Colorectal Carcinogenesis

**Figure 2:**
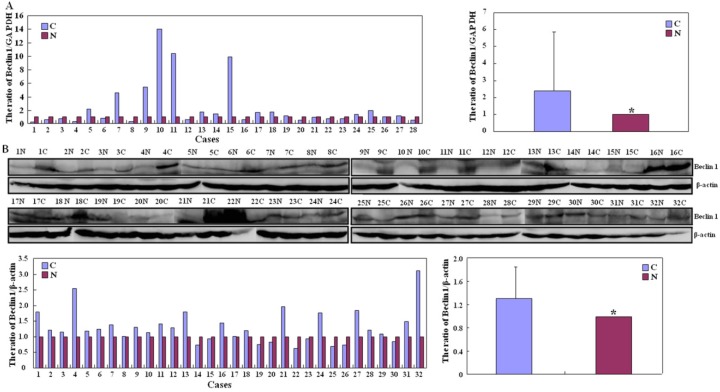
Beclin 1 expression in colorectal carcinoma and matched non-neoplastic mucosa (**A**) Quantification of *Beclin 1* mRNA was performed in colorectal carcinoma and non-neoplastic mucosa (NNM) by real-time RT-PCR. *Beclin 1* mRNA levels were significantly higher in colorectal carcinoma than paired mucosa (* *p* < 0.05); (**B**) Tissue lysate was loaded and probed with anti-Beclin 1 antibody (60 kDa) with β-actin (42 kDa) as an internal control. Densitometric analysis showed higher Beclin 1 expression in carcinoma than matched mucosa (* *p* < 0.05). Note: N, non-neoplastic mucosa; C, carcinoma.

**Figure 3:**
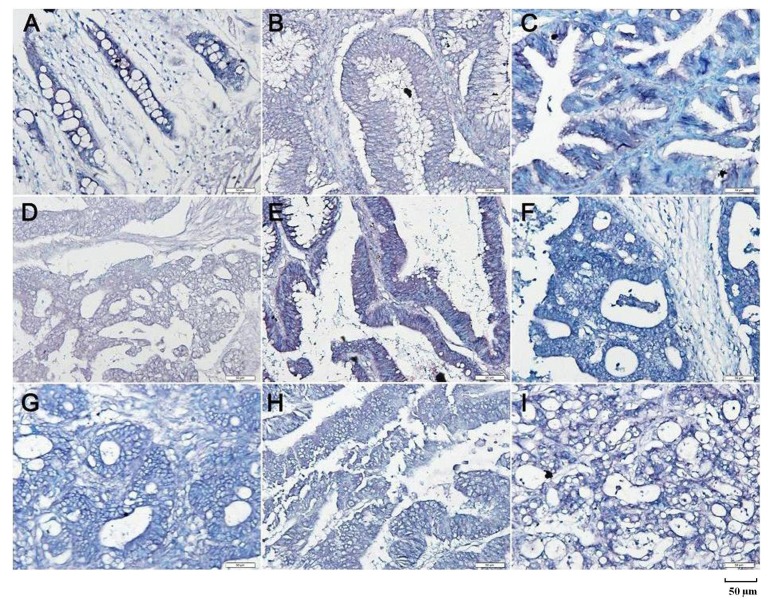
*Beclin 1* mRNA expression in colorectal carcinogenesis by* in situ* hybridization. *Beclin 1* mRNA was observed in the cytoplasm of the colorectal non-neoplastic epithelial cells (**A**); adenoma (**B**,**C**); primary adenocarcinomas (**D**,**E**) and metastatic carcinoma in lymph node (**F**,**G**) and liver (**H**,**I**).

**Figure 4:**
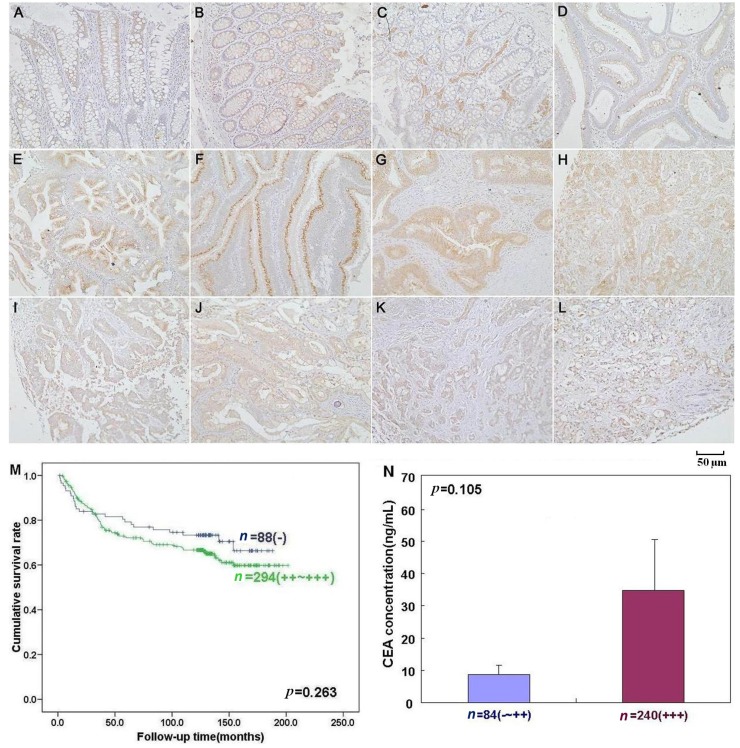
Beclin 1 protein expression and its correlation with prognosis and CEA (carcinoembryonic antigen) concentration of colorectal carcinoma by immunohistochemistry. Beclin 1 was positively expressed in the cytoplasm of colorectal epithelial cells (**A**–**C**), macrophages (**C**), infiltrating inflammatory cells (**A**–**C**) of colorectal mucosa (**D**), colorectal adenoma (**E**), well (**F**)-, moderately (**G**)-, and poorly (**H**)-differentiated carcinoma, metastatic carcinomas in lymph node (**I**,**J**) and liver (**K**,**L**). *Kaplan-Meier* curves for cumulative survival rate of patients with overall colorectal carcinomas according to Beclin 1 expression (**M**). There was no link between serum CEA and Beclin 1 expression in the colorectal carcinoma patients (**N**, *p* > 0.05).

*Beclin 1* mRNA expression was significantly greater in carcinoma than that in adjacent NNM by real-time PCR (*p* < 0.05, Figure 2A). There was higher intensity of Beclin 1 protein bands in carcinoma than paired mucosa by Western blot (*p* < 0.05, Figure 2B). According to the* in situ* hybridization (ISH) data, *Beclin 1* mRNA expression was lower in colorectal non-neoplastic mucosa (NNM) than adenoma and carcinoma (*p* < 0.05, Figure 3 and Table 1). As indicated in Figure 4, Beclin 1 protein was expressed in the cytoplasm of colorectal superficial mucosa, macrophages, infiltrating inflammatory cells, tubular and villous adenoma, primary and metastatic carcinomas by immunohistochemistry. In the present study, Beclin 1 expression in colorectal carcinoma, adenoma and NNM was considered for statistical analysis. Primary carcinoma showed stronger Beclin 1 expression than NNM and metastatic carcinoma in the liver (*p* < 0.05, Table 1). Taken together, Beclin 1 overexpression was found during colorectal carcinogenesis.

**Table 1:** Beclin 1 expression in colorectal carcinogenesis and metastatic foci.

**Groups**	***n***	***Beclin 1* mRNA Expression **					***n***	**Beclin 1 Protein Expression**				
		−	+	++	+++	%		−	+	++	+++	%
Non-neoplastic mucosa	74	39	24	9	2	47.3 *	550	281	112	108	49	48.9 *
Adenoma	101	9	11	45	36	91.1	139	5	29	61	44	96.4
Primary carcinoma	106	26	31	24	25	75.5	589	50	100	158	281	91.5
Metastatic carcinoma in lymph node	51	5	18	23	5	90.2	195	17	28	65	85	91.3
Metastatic carcinoma in liver	26	9	5	8	4	65.4	48	17	11	9	11	64.6 ^†^

%, positive rate; * *p* < 0.001, compared with adenoma and carcinoma; ^†^ *p* < 0.05, compared with primary carcinoma.

### 2.3. The Close Relationship between Beclin 1 Expression and Aggressiveness of Colorectal Carcinoma

As summarized in Table 2, immunostaining level of Beclin 1 protein was negatively related to liver and distant metastasis (*p* < 0.05), but not correlated with age, sex, depth of invasion, lymphatic or venous invasion, lymph node metastasis, tumor-node-metastasis (TNM) staging, differentiation or serum carcinoembryonic antigen (CEA) concentration (*p* > 0.05), indicating that Beclin 1 hypoexpression was employed to reflect late progression of colorectal carcinoma. Follow-up information was available from 382 patients with colorectal carcinoma (CRC) for periods ranging from 1.6 months to 16.8 years (median = 10.5 years). Univariate analysis using *Kaplan-Meier* method showed no correlation between Beclin 1 protein expression and cumulative survival rate of the patients with CRC despite stratification to depth of invasion (*p* > 0.05, Figure 4). Multivariate analysis using Cox’s proportional hazard model indicated that depth of invasion and distant metastasis were independent prognostic factors for overall CRCs (*p* < 0.05, Table 3).

**Table 2:** Relationship between Beclin 1 protein expression and clinicopathological features of colorectal carcinomas.

**Clinicopathological Features**		***n***	**Beclin 1 Protein Expression**					
			**−**	**+**	**++**	**+++**	**%**	***p Value***
Age (years)	＜65	286	22	45	82	137	92.3	0.587
	≥65	303	28	55	76	144	90.8	
Sex	Male	343	31	64	96	152	91.0	0.052
	Female	246	19	36	62	129	92.3	
Depth of invasion	T_is_-T_2_	137	8	18	44	67	94.2	0.323
	T_3_-T_4_	433	39	77	111	206	91.0	
Lymphatic invasion	−	224	19	38	57	110	91.5	0.471
	+	284	26	50	79	129	90.8	
Venous invasion	−	243	21	52	66	104	91.4	0.084
	+	251	23	35	67	126	90.8	
Lymph node metastasis	−	306	24	54	86	142	92.2	0.719
	+	261	23	44	66	128	91.2	
Liver metastasis	−	528	42	88	137	261	92.0	0.003
	+	54	8	11	20	15	85.2	
Distant metastasis	−	523	42	87	136	258	92.0	0.017
	+	62	8	12	22	20	87.1	
TNM staging	I–II	243	14	40	67	122	94.2	0.822
	III–IV	234	20	32	59	123	91.5	
Differentiation	Well-differentiated	260	21	46	78	115	91.9	0.433
	Moderately-differentiated	269	22	43	63	141	91.8	
	Poorly-differentiated	35	4	7	10	14	88.6	

%, positive rate; T_is_ = carcinoma* in situ*; T_1_ = lamina propria and submucosa; T_2_ = muscularis propria; T_3_ = subserosa and exposure to serosa; T_4_ = invade other organs or perforate visceral peritoneum; %, positive rate.

**Table 3:** Multivariate analysis of clinicopathological variables for the survival of the patients with colorectal carcinomas.

Clinicopathological Parameters	Relative Risk (95% CI)	*p* Value
Age (≥65 years)	0.823 (0.480–1.411)	0.478
Sex (female)	0.870 (0.535–1.412)	0.572
Depth of invasion (T_2-4_)	7.086 (2.122–23.657)	0.001
Lymphatic invasion (+)	1.391 (0.790–2.449)	0.252
Venous invasion (+)	0.983 (0.551–1.751)	0.953
Lymph node metastasis (+)	1.434 (0.415–4.953)	0.569
Liver metastasis (+)	0.360 (0.069–1.867)	0.224
Distant metastasis (+)	11.490 (2.464–53.573)	0.002
TNM staging (III–IV)	1.991 (0.491–8.063)	0.335
Differentiation (poorly-differentiated)	1.221 (0.820–1.819)	0.325
Beclin 1 protein expression (+++)	1.338 (0.837–2.136)	0.223

CI = confidence interval; TNM = tumor-node-metastasis.

### 2.4. Discussion

Beclin 1 protein was found to mainly localize in the cytoplasm of carcinoma cell lines, the superficial mucosa, adenoma, infiltrating inflammatory cells, and primary and metastatic carcinoma, suggesting that the expression pattern of Beclin 1 has cellular specificity, thus determining its biological functions. In contrast to other findings in cutaneous squamous cell carcinoma [19] and pancreatic ductal adenocarcinoma [20], Beclin 1 protein expression was higher in CRC than colorectal adenoma and adjacent NNM which is in line with gastric [21] and ovarian cancers [22], suggesting that Beclin 1 overexpression might play an important role in colorectal adenoma-adenocarcinoma sequence as an early event. Our Western blot analysis also showed the same results in gastric [21] and ovarian [22] carcinomas although Beclin 1 expression in stromal cells from colorectal samples could be excluded from immunohistochemical data by their specific histomorphological features and topographic location. According to the results of real-time RT-PCR and ISH, *Beclin 1* mRNA level was higher in CRC than paired NNM, consistent with the previous reports about intrahepatic cholangiocellular carcinoma [23], and hepatocellular cell carcinoma [24]. These data indicate that up-regulated Beclin 1 expression is closely associated with the malignant transformation of colorectal epithelial cells. Here, Beclin 1 overexpression might be a result for colorectal carcinogenesis and function as tumor suppressor. To confirm the hypothesis, we are conditionally deleting the *Beclin 1* gene in intestinal mucosa and will observe the pathomorphological alteration of intestinal epithelium in the future.

In agreement with the report about pancreatic ductal adenocarcinoma [20], Beclin 1 expression was negatively linked to liver and distant metastasis of colorectal carcinomas. Compared with primary carcinoma, Beclin 1 expression was decreased in metastatic carcinoma into the liver, which might be attributed to the low Beclin 1 expression in colorectal carcinoma with liver metastasis. It was suggested that Beclin 1 overexpression might suppress the metastasis of CRC and Beclin 1 hypoexpression could be employed to indicate late progression of CRC. The* in vitro* experiment showed that Beciln 1 overexpression could suppress the migration and invasion of colorectal carcinoma cells (data not published). It has been reported that decreased Beclin 1 expression is significantly associated with lymph node metastasis of intrahepatic cholangiocellular [23], cervical [13], ovarian [25] and esophageal squamous cell [26] carcinomas. Our data also demonstrated that Beclin 1 expression was inversely associated with venous invasion, lymph node metastasis, and TNM staging of gastric carcinoma [21]. Pirtoli* et al.* [27] found that Beclin 1 expression was positively correlated with apoptosis, and negatively with cell proliferation in high-grade glioma. Koneri* et al.* [28] transfected *Beclin1* gene into HT29 colon cancer cells and found that ectopic Beclin 1 overexpression resulted in slow cell growth and G_1_ arrest with the down-regulated expression of cyclin E and phosphorylated Rb. In combination of these findings, it is suggested that Beclin 1 overexpression might reverse the aggressive phenotypes as a tumor suppressor in colorectal cancers.

Here, there was no link between Beclin 1 expression and survival of CRC patients, which differs from results of pancreatic ductal adenocarcinoma [20], intrahepatic cholangiocellular carcinoma [23], esophageal squamous cell carcinoma [26], stage IIIB colon carcinoma [29], and nasal-type extranodal natural killer T-cell lymphoma [30]. Previously, Beclin 1 expression was found to be positively correlated with favorable prognosis of patients with overall and intestinal-type carcinoma as an independent factor [21]. In ovarian carcinoma, Beclin 1 can be considered a prognostic factor, but not an independent factor [22]. In contrast, Wan* et al.* [31] found that nasopharyngeal carcinoma patients with lower Beclin 1 expression displayed a significant overall survival advantage than those patients with higher Beclin 1 expression. Multivariate analysis demonstrated that depth of invasion and distant metastasis were independent factors for CRCs. Taken together, the prognostic significance of Beclin 1 protein expression is of cancer specificity. Our data indicated that Beclin 1 expression could not be employed to evaluate the survival status of CRC patients.

## 3. Experimental Section

### 3.1. Cell Culture

Colorectal carcinoma cell lines were gifts from Professor Sugiyama, Department of Gastroenterology, University of Toyama, Japan and Professor Miyagi, Clinical Research Institute, Kanagawa Cancer Center, Japan. The cell lines were grown in RPMI 1640 (Colo201, Colo205, DLD-1. HCT-15, HCT-116, HT-29, KM-12, SW-480, and SW-620) and Dulbecco’s modification of Eagle’s medium (DMEM, WiDr) medium supplemented with 10% fetal bovine serum (FBS), 100 units/mL penicillin, and 100 μg/mL streptomycin in a humidified 5% CO_2_ at 37 °C. All cells were harvested by centrifugation and rinsed with phosphate buffered saline (PBS).

### 3.2. Immunofluorescence Staining

Cells were seeded on glass coverslips until adhesion and fixed with 4% formaldehyde in PBS for 10 min, and permeabilized with 0.2% Triton X-100 in PBS for 10 min at room temperature. After washing with PBS, cells were blocked with 1% bovine serum albumin for 30 min and subsequently incubated overnight at 4 °C with rabbit anti-Beclin 1 (Sigma, St. Louis, MI, USA). Following wash by PBS, the slides were incubated with the anti-goat IgG-Alexa 576 (Invitrogen, New York, NY, USA) antibody at room temperature for 1 h. The cell nuclei were stained with 1 μg/mL DAPI (Sigma) for 30 min at 37 °C. Finally, coverslips were mounted with SlowFade^®^ Gold antifade reagent (Invitrogen) and observed using laser confocal microscope (Olympus, Tokyo, Japan).

### 3.3. Subjects

Colorectal carcinomas (*n* = 589, CRC), adjacent non-neoplastic mucosa (NNM, *n* = 550), adjacent adenoma (*n* = 139), lymph nodes (*n* = 195) and livers (*n* = 48) with metastasis were collected from surgical resection in the Affiliated Hospital of Kanagawa Cancer Center. The patients with colorectal carcinoma included 343 men and 246 women (26~86 years, mean = 64.4 years). We also collected fresh colorectal carcinoma and adjacent NNM in the First Affiliated Hospital of China Medical University and frozen in −80 °C. No patients underwent chemotherapy, radiotherapy or adjuvant treatment prior to surgery. All patients provided written consent for the use of the tumor tissue for clinical research. The Ethical Committee of our University and Kanagawa Cancer Center approved the research protocol and sample collection. Patients were followed up by consulting their case documents and by telephone contact.

### 3.4. Pathology

All tissues were fixed in 10% formalin, embedded in paraffin and cut into 4 μm thick sections. Sections were stained with Harris hematoxylin-and-eosin (HE) to confirm histological characteristics. The staging of human colorectal carcinoma was evaluated according to the Union Internationale Contre le Cancer (UICC) system [32]. Histological architecture of CRCs was expressed in terms of the World Health Organization (WHO) classification [33]. Furthermore, tumor size, depth of invasion, lymphatic and venous invasion were determined.

### 3.5. Reverse Transcriptase-Polymerase Chain Reaction (RT-PCR)

Total RNA was isolated from colorectal carcinoma cells using QIAGEN RNeasy mini kit (QIAGEN, Hilden, Germany). The first strand cDNA synthesis was performed using Avian myeloblastosis virus (AMV) reverse transcriptase and random primer (Takara, Otsu, Japan). Oligonucleotide primers for PCR are Forward: 5'-GATGGAAGGGTCTAAGACGTCCAA-3' and Reverse: 5'-TTTCGCCTGGGCTGTGGTAAG-3' (NM_003766.3, 162−321, 160 bp) for Beclin 1 and Forward: 5'-CAATGACCCCTTCATTGACC-3' and Reverse: 5'-TGGAAGA TGGTGATGGGATT-3' (NM_002046.3, 201–335, 135 bp) for GAPDH. General and real-time RT-PCR amplification of cDNA was performed using Hotstart Taq polymerase and SYBR Premix Ex Taq™ II kit (Takara) respectively.

### 3.6. Western Blot (WB)

The cells were lysed in radio-immunoprecipitation assay (RIPA) buffer (50 mM Tris-HCl pH 7.5), 150 mM NaCl, 5 mM Ethylene Diamine Tetraacetie Acid (EDTA), 0.5% Nonidet P-40 (Sigma), 5 mM dithiothreitol, 10 mM NaF, protease inhibitor cocktail (Nacalai tesque, Tokyo, Japan). The protein was determined by protein assay (Biorad, Berkeley, CA, USA), separated on a Sodium dodecyl sulfate (SDS)-polycrylamide gel and transferred to Hybond membrane (Amersham, Amersham, Germany). The membrane was then blocked overnight in 5% skim milk in Tris-buffered saline with Tween 20 (TBST). For immunoblotting, the membrane was incubated with the anti-Beclin 1 (Sigma). The membrane was rinsed with TBST and incubated with anti-rabbit, anti-goat or anti-mouse IgG conjugated to horseradish peroxidase (DAKO, Carpinteria, CA, USA). Protein bands were visualized with X film by ECL-Plus detection reagents (Santa Cruz, CA, USA). The membranes were washed with WB Stripping Solution (Nacalai) and treated as described above except for anti-β-actin antibody (Santa Cruz).

### 3.7. Tissue Microarray (TMA) and in Situ Hybridization (ISH)

Under the guidance of HE stained sections, the 2 mm-in-diameter tissue cores per donor block were removed and transferred to a recipient block using a Tissue Microarrayer (AZUMAYA, Tokyo, Japan). To perform RNA-DNA ISH for *Beclin 1*, a digoxygenin-labeled Beclin 1 probe was made using the above-mentioned primers and HCT-15 cDNA by RT-PCR. The sections of TMA were deparaffinized and digested with proteinase K at 37 °C. Twenty μL of probe in hybridization buffer was added to each slide. After coverslipping, heating, and overnight incubation in a humidified chamber at 42 °C, sections were rinsed in TBST and incubated with anti-digoxygenin antibody conjugated with alkaline phosphatase (Roche, Hongkong, China) at 37 °C. The slides were then washed and immersed in solution II (100 mM Tris-HCl pH 9.5, 100 mM NaCl and 50 mM MgCl_2_) followed by exposure to NBT(nitro-blue tetrazolium chloride)/BCIP (5-Bromo-4-Chloro-3'-Indolylphosphatase *p*-Toluidine salt) as a chromogen. Finally, counterstaining was performed using methyl green. As indicated in Figure 3, *Beclin 1* mRNA signal was positively stained in the cytoplasm (Figure 3) and 100 cells were randomly selected and counted from 5 representative fields of each section. Uncertain data were blindly confirmed by two independent observers (Zhang M.Y. and Zheng H.C.) until a final agreement was reached. The expression level was graded and scored as follows: 0 = negative; 1 = 1%–50% positive staining; 2 = 50%–74% positive staining; 3 ≥ 75% positive staining.

### 3.8. Immunohistochemistry (IHC)

Consecutive sections of TMA were deparaffinized and subjected to antigen retrieval by irradiation in target retrieval solution (TRS, DAKO) in a microwave oven. The sections were quenched with 3% H_2_O_2_ in methanol to block endogenous peroxidase. Five percent bovine serum albumin (BSA) was then applied to prevent non-specific binding. The sections were incubated with rabbit anti-Beclin 1 (Sigma), then treated with the rabbit conjugated to horseradish peroxidase (DAKO) antibodies. All the incubations were performed in a microwave oven to allow intermittent irradiation as previously described [34]. After each treatment, the slides were washed with TBST three times. The immunoreactivity was visualized with 3,3'-diaminobenzidine (DAB). Finally, the sections were counterstained with Mayer’s hematoxylin, dehydrated, and mounted with coverslips. Omission of the primary antibody was used as a negative control.

As indicated in Figure 4, Beclin 1 protein was positively localized in the cytoplasm. The expression positivity was graded and scored as follows: 0 = negative; 1 = 1%–50% positive staining; 2 = 50%–74% positive staining; 3 ≥ 75% positive staining. The staining intensity score was graded as follows: 1 = weak; 2 = intermediate; 3 = strong. The scores for Beclin 1 positivity and staining intensity were multiplied to obtain a final score, thus determining its expression as: − = 0; + = 1–2; ++ = 3–5; +++ = 6–9.

### 3.9. Measurement of Carcinoembryonic Antigen (CEA)

Serum CEA was determined using Chemiluminescence Immunoassay (Diagnostic Agnostic Automation Inc., Tokyo, Japan). Briefly, 50 μL of standard (0–120 ng/mL), specimens, and controls were dispensed into the appropriate wells. Then, 100 μL of enzyme conjugate reagent was added to each well, the plate gently mixed and incubated. The microtiter wells were rinsed with wash buffer. Residual water droplets were removed by striking the well sharply onto absorbent paper. Finally, 100 μL Chemiluminescence substrate solution was added to each well, mixed gently and subjected to absorbance determination.

### 3.10. Statistical Analysis

Statistical evaluation was performed using the Spearman correlation test to analyze the rank data, and Mann-Whitney *U* test to differentiate the means of different groups.* Kaplan-Meier* survival plots were generated and comparisons made with the log-rank statistic. Cox’s proportional hazards model was employed for multivariate analysis. A *p* value of *p* *<* 0.05 was considered statistically significant.

## 4. Conclusions

In summary, Beclin 1 expression may impact the malignant transformation of colorectal epithelial cells and could be considered a good biomarker for colorectal carcinogenesis and distant metastasis.
